# 

**Published:** 1868-05

**Authors:** 


### CONTENTS.

ORIGINAL COMMUNICATIONS.

Local Hyperemia, or Excess of Blood in Dental Tissues ..... 179
Is the Sense of Taste impaired by Plates? ..... 185
Six Year Molars ..... 189
Microscopy of the Teeth, continued ..... 191

PROCEEDINGS OF SOCIETIES.

Proceedings of the Association of the Colleges of Dentistry ..... 197
Proceedings of the Northern Ohio Dental Association ..... 202
Indiana State Dental Association ..... 209

SELECTIONS.

Materia Medica, General Therapeutics and Pharmacy ..... 210
Microscopy of the Teeth ..... 214

EDITORIAL.

Ohio State Dental Society ..... 219
A Practical Treatise on Operative Dentistry ..... 220
Suggestive ..... 221
Notice ..... 222
American Dental Association ..... 222

